# The Potential of Antimicrobial Peptides as Biocides

**DOI:** 10.3390/ijms12106566

**Published:** 2011-10-06

**Authors:** Garry Laverty, Sean P. Gorman, Brendan F. Gilmore

**Affiliations:** 1 Ward Biotech Ltd., Glasdrummann, Milltown, Monaghan, Ireland; 2 Biomaterials Research Group, School of Pharmacy, Queens University of Belfast, Medical Biology Centre, 97 Lisburn Road, Belfast BT9 7BL, UK; E-Mails: s.gorman@qub.ac.uk (S.P.G.); b.gilmore@qub.ac.uk (B.F.G.)

**Keywords:** antimicrobial, peptides, biocides

## Abstract

Antimicrobial peptides constitute a diverse class of naturally occurring antimicrobial molecules which have activity against a wide range of pathogenic microorganisms. Antimicrobial peptides are exciting leads in the development of novel biocidal agents at a time when classical antibiotics are under intense pressure from emerging resistance, and the global industry in antibiotic research and development stagnates. This review will examine the potential of antimicrobial peptides, both natural and synthetic, as novel biocidal agents in the battle against multi-drug resistant pathogen infections.

## 1. Introduction

### 1.1. Antimicrobial Peptides

Despite continuing efforts, the increasing prevalence of resistance among pathogenic bacteria to common antibiotics has become one of the most significant concerns in modern medicine. With significantly reduced investment in antimicrobials research and development among major pharmaceutical companies, novel alternatives to existing treatment strategies are not being produced at a sufficient rate to keep pace with the emergence of resistance and the supply pipeline runs perilously close to drying up [1]. Incidences of hospital-acquired and community-acquired antibiotic resistant *Staphylococcus aureus* infections have risen dramatically in recent years [2], with almost 50% of hospital acquired *Staphylococcus aureus* infections classified as methicillin resistant and 30% of enterococci exhibiting vancomycin resistance [3]. In 2010, the Infectious Diseases Society of America launched its 10 × 20 initiative, calling for a global commitment to new antibacterial drug development, with the goal of ten new antibiotic agents by the year 2020 [4]. In addition, continued extensive use of the limited classes of effective antibiotics currently available threatens to call time on the ‘antibiotic era’. In the past decade, the crisis of antimicrobial resistance has worsened significantly and there exists an urgent requirement for new antimicrobial agents with activity against multidrug resistant pathogens [5].

One area of antimicrobial drug research that does shows significant promise is in the discovery and development of antimicrobial peptides (AMPs). Antimicrobial peptides in nature serve as important defensive weapons throughout the animal and plant kingdoms against a broad spectrum of bacterial and fungal pathogens [6]. Sources range from single celled microorganisms, such as bacteria themselves (bacteriocins) [7], to invertebrates [8]. As well as having a direct effect on the microorganism, antimicrobial peptides have been proven to promote the accumulation of immune cells including macrophages, neutrophils and lymphocytes [9]; neutralize lipopolysaccharide endotoxin derived from Gram-negative bacteria [10]; aid wound repair; stimulate angiogenesis [11] and control the actions of the innate and adaptive immune response with little or no resistance development reported. Antimicrobial peptides exert their microbicidal effect via disruption of the microbial cell membrane together with intracellular action [12,13].

Antimicrobial peptides are short (typically ranging from 12–100 amino acid residues in length), exhibit rapid and efficient antimicrobial toxicity against a range of pathogens [14,15] and constitute critical effector molecules in the innate immune system of both prokaryotic and eukaryotic organisms [16]. To date, over 1700 endogenous antimicrobial peptides have been isolated with many more synthetic analogues reported in the literature [17]. Structure activity relationship analyses have yielded vital information relating to the structural features of effective antimicrobial peptides, indicating that antimicrobial activity is governed primarily by charge and hydrophobicity [18], and that the initial target is the negatively charged bacterial cell membrane [19]. These studies have also facilitated the design of ultrashort, highly active antimicrobial peptide scaffolds [20,21] which may be prepared via established facile, solid phase synthetic protocols at lower costs compared with their natural antimicrobial peptide counterparts [22,23].

### 1.2. Cationic Antimicrobial Peptides

The majority of antimicrobial peptides are cationic with more than a thousand characterized and are thus termed cationic antimicrobial peptides (CAPs) [24]. Naturally derived cationic antimicrobial peptides typically consist of a net positive charge between +2 and +9, due to the presence of few or no acidic residues, such as glutamate or aspartate and a high number of cationic amino acids such as lysine or arginine and/or histidine [25]. Hydrophobic residues, including tryptophan and branched amino acids such as valine, form 30–50% of the total peptide structure and serve a vital role in allowing a typical amphiphilic structure to form upon interaction with membranes [26]. This characteristic together with the presence of dense areas of high positive charge allow cationic antimicrobial peptides to exert their antimicrobial effect. Alteration of this net charge to hydrophobic ratio can vary the activity and spectrum of the peptide against a host of microorganisms. The increase in activity that can be therapeutically achieved by tapering the lipophilic:charge ratio is provided by the example of glycopeptides. Glycopeptides, for example vancomycin, represent one of the last line of effective antibiotics against Methicillin resistant *Staphylococcus aureus*. Lipoglycopeptide derivatives of vancomycin in development include oritavancin and dalbavancin which possess increased activity against vancomycin-resistant strains [27]. Slight modifications in the balance of hydrophobicity and charge can give rise to marked changes in antimicrobial selectivity/activity [28]. Cationic antimicrobial peptides, as a class of compounds, have broad structural diversity and antimicrobial spectrum influenced primarily by the amino acids that constitute the primary sequence of the peptide [29].

Although the secondary structures that antimicrobial peptides adopt may vary between classes they share the same characteristic of developing an amphipathic structure and being cationic under physiological conditions [30,31]. Secondary structures include amphiphilic β-sheet structures containing two or three stabilizing disulphide bonds, often with a short α-helical segment and/or two to four β-strands. These disulphide bridges form as a result of a cysteine-rich primary sequence. Examples of these peptides in nature include several classes of mammalian host defense peptides including α-defensin and β-defensin [32]. Linear peptides are unordered in hydrophilic solutions but form amphipathic α-helices upon contact with cell membranes and in a hydrophobic environment [33].

These peptides are of particular interest as one face of the helix structure contains a majority of hydrophobic residues whereas the opposite face contains mainly polar amino acids allowing efficient solubilization of microbial membranes [26]. Amphipathic α-helices lack cysteine and are thus unable to form disulphide bridges [34]. Examples in nature include mellitin derived from honeybee venom [35], magainin obtained from the skin secretions of the frog species *Xenopus laevis* [36] and the cecropins, a group of the dipteran insect defense peptides [37]. Cyclic peptides are a less prevalent structural class of cationic antimicrobial peptides that contain β-turn influenced by a single disulphide bond and include a dodecapeptide from bovine neutrophils [38].

Extended structures with a high regularity of one or two amino acid residues, such as proline, glycine, histidine or tryptophan, make up the remainder of the four major structural varieties [15,39]. Extended cationic peptides have no defined or typical structure due to the existence of novel folds. Examples in nature include the porcine derived tritrpticin [40] and the bovine neutrophil peptide indolicidin [41]. Indolicidin is a linear antimicrobial peptide consisting of a 13 amino acid structure high in tryptophan residues (~40%) [42]. Both these peptides form boat-like structures in the presence of cell membranes as the high tryptophan content interacts with the hydrophobic layer of the membrane with the remaining cationic arginine and lysine residues orientated towards the aqueous environment [43]. The cationic AMP mimetic tyrocidin was the first commercially available antibiotic. However major issues with toxicity toward human blood and reproductive cells lead to its withdrawal from the market [44]. The polypeptide bacitracin has had a more successful introduction clinically incorporated with both neomycin and polymyxin B in the topical product Neosporin® licensed for the topical treatment of a variety of localized skin and eye infections [45].

### 1.3. Anionic Antimicrobial Peptides

Despite the vast majority of antimicrobial peptides being cationic in nature, a significant number of anionic AMPs have been reported; serving as important weapons in the eukaryotic innate immune response [46–48]. Peptides that are anionic in nature tend to be rich in glutamic and aspartic acids and include the amphibian peptide Maximin-H5 and Dermcidin, a peptide derived from human sweat [49,50]. Anionic antimicrobial peptides commonly consist of 5 to 70 amino acid residues, possessing a net charge of −1 or −2 although structural characterization demonstrated that the truncated form of bovine peptide B, termed enkelytin, can possess a net charge as high as −7 [48,51]. Although less common, anionic peptides of 300 residues in length and net charge −20 have been reported [52–54]. Similarly to their cationic counterparts, anionic antimicrobial peptides can adopt varying amphiphilic structures such as the α-helix and the β-sheet conformations with interaction with the microbial membrane key to activity.

A disadvantage of many anionic antimicrobial peptides is that they often require cations, for example zinc (Zn^2+^), as cofactors for biocidal activity [55]. This may be why they are generally located at epithelia; sites were both ionic secretion and microbial susceptibility are highest. Anionic peptides, for example surfactant-associated anionic peptides present in pulmonary tissue, have been shown to possess increased potency against both Gram-positive and Gram-negative bacteria in the presence of synergistic cationic antimicrobial peptides and Zn^2+^ [56,57]. These cationic moieties act as a cationic linkage between the anionic antimicrobial peptide and the anionic microbial cell membrane. This allows transport of the anionic peptide to intracellular targets without damaging to the structure of the microbial membrane [55,58]. Anionic peptides target ribosomes within the cell inhibiting ribonulease activity, thus resulting in microbial cell death [59,60].

### 1.4. Amphibian Antimicrobial Peptides

Amphibian derived peptides are excellent examples of naturally occurring, structurally diverse peptides with high antimicrobial potency. These compounds are released in skin secretions often at high concentrations and their production reflects the evolution of amphibians to their humid habitat, an environment also suitable for the growth and proliferation of opportunistic pathogenic bacteria and fungi [61]. Such peptides are also beginning to show promise against cancer, as anti-tumor compounds [62] and also possess anti-viral activity, with potential benefits against HIV [63]. They also show activity against eukaryotic cells and therefore provide a means by which amphibians may be protected from predation [64]. The potential therefore exists that amphibian derived antimicrobial peptides may also be cytotoxic to humans. Such peptides are released naturally in response to injury or stress via contraction of myocytes surrounding the glands [65] and this may be replicated in the laboratory through the use of mild electrical stimulation [66] or injecting norepinephrine into the dorsal sac [67].

Structural characterization has facilitated their synthetic production *via* solid phase peptide synthesis (SPPS). In frogs, variations in the sequence and spectrum of activity of these peptides are considerable with hundreds of characterized peptides providing protection against a vast range of bacterial and fungal species [61,68,69]. The majority of antimicrobial peptides from frogs are cationic due to a high presence of lysine, with at least 50% of amino acids being hydrophobic of which leucine is most prevalent and an amphipathic α-helix secondary structure predominates at the cell membrane interface [61,70]. Relevant genera of frog studied include *Bombina* [71], *Xenopus* [36], *Rana* [72], *Phyllomedusa* [73] and *Litoria* [74]. The species *Bombina maxima*, commonly known as the Chinese red belly toad has as many as forty genes linked to the production of different antimicrobial peptides [75]. Research conducted by Lai *et al.* showed that *Bombina maxima* produced a group of peptides called maximins that demonstrated minimum inhibitory concentration (MIC) values in the μg/mL range against a broad spectrum of microbial and fungal pathogens including *Staphylococcus aureus*, *Escherichia coli*, *Bacillus dysenteriae*, *Klebsiella pneumoniae* and *Candida albicans* [76]. Both maximin-3 and maximin-4 were the most potent peptides tested with maximin-4 having the lowest MIC value of 2.7 μg/mL against *Staphylococcus aureus*. Maximin-4 consists of twenty seven amino acids and has the potential to be produced synthetically *via* SPPS. Dermaseptins are a family of linear lysine-rich cationic antimicrobial peptides derived from the genus *Phyllomedusa*. They consist of between twenty eight and thirty four amino acid residues and have been shown to inhibit the growth of a wide variety of Gram-positive and Gram-negative bacteria, fungi, protozoa and yeast [73,77].

### 1.5. Rational Design and Selection of an Antimicrobial Peptide Motif

The diverse range of antimicrobial peptides available in nature provides a vast scope for the design of novel and improved synthetic variations. Structure and its effect on the hydrophobic:charge ratio is more important with regard to antimicrobial activity than size and the manipulation of primary amino acid sequence can improve factors such as specificity, toxicity and stability [12,78]. Ultrashort antimicrobial peptides consist of approximately four or five amino acids residues, with amino acid selection fulfilling the minimum range of functionalities required for effective antimicrobial activity. These functionalities include a charged moiety such as arginine and a lipophilic unit, most commonly tryptophan, forming an antimicrobial pharmacophore with the correct balance between charge and lipophilicity [79].

Strom *et al.* have proven that tryptophan provides better bulk and lipophilicity than tyrosine and a minimum of two bulk and two charged residues are required to be present to give activity against staphylococci, with *Escherichia coli* requiring an additional bulk tryptophan. Arginine also acts a better source of charge than lysine within the minimum motif [78]. However arginine is difficult to work with in terms of SPPS [80,81]. The obvious advantage to the use of ultrashort antimicrobial peptide is the large reduction in cost in synthesizing these molecules relative to synthetic variants of naturally occurring antimicrobial peptides. The attachment of an acyl chain to an active or inert ultrashort cationic peptide also potentially leads to an increased action against microorganisms in a similar way to native cationic antimicrobials [82]. Small alterations of the total methylene units present can also modify the spectrum of activity for the antimicrobial, the cell specificity and therefore levels of hemolysis [83]. Previous studies by Makovitski *et al.* reported however that peptides containing four amino acids would find it difficult to form a characterized, stable amphipathic structure, therefore questions still relate to their mode of action. Reported results also show that the balance of hydrophobicity and charge play an important role just as in the larger antimicrobial peptides. By altering both, the amino acid residues and the acyl chain length, it was found that increasing hydrophobicity and/or charge is not necessarily indicative to increasing antimicrobial function [84].

Host and microbial proteases and the unfavorable pharmacokinetics they bring present a major barrier to the use of peptides as antimicrobials *in vivo*, with many peptides limited to use as topical applications. Human chymotrypsin-like enzymes function at basic amino acid residues [85], therefore antimicrobial peptides are a viable target due to the requirement of basic residues to be present for antimicrobial activity. Simple α-helical or linear structures in particular are susceptible to proteolysis by a range of microbial proteases [86]. As proteases will only recognize natural L-amino acid residues, a switch to the use of D-amino acids at susceptible points will render the peptide partially or totally resistant to proteolysis without loss of antimicrobial activity [87]. L-stereoisomers also tend to be more hemolytic than their D counterparts [88]. Antimicrobial peptides are expensive to synthesize and costs are increased further by the use of d-enantiomers thus a more viable alternative is to select unnatural amino acids, such as ornithine. The use of ornithine as a charged moiety is preferable as the use of a non-coded amino acid provides stability against proteases [78]. Examples of synthetic harnessing of ornithine’s properties are demonstrated by Bisht *et al*. They demonstrated the potent action of ultrashort tetrapeptides with two ornithines representing the charged portion and two tryptophans providing bulk and hydrophobicity [21]. Modification occurred with conjugation of cinnamic groups to the N^α^-amino terminus of ornithine. The incorporation of ornithine into the lipopeptide scaffold has also been tested in the synthetic peptide MSI-843 [89]. Containing six ornithine residues out of a total ten and a conjugated octanyl terminus, excellent activity was shown against Gram-positive *Staphylococcus aureus*; Gram-negative *Pseudomonas aeruginosa* and *Escherichia coli* and the fungus *Candida albicans*.

### 1.6. Ultrashort Cationic Antimicrobial Peptides

Ultrashort cationic antimicrobial peptides consist of approximately four or five amino acids residues, with amino acid selection fulfilling the minimum range of properties required for effective antimicrobial activity to take place. The use of ultrashort cationic antimicrobial peptides is advantageous in terms of manufacture with reduced production costs and synthesis times. Their production correlates to a trend amongst peptide companies that has seen an increase in the production of these smaller peptides in relation to larger, more expensive natural peptides [90,91]. Haug and colleagues discovered, using lactoferrin derivatives, that a minimum motif existed for antibacterial action against many forms of bacteria including strains of *Staphylococcus aureus* resistant to common antibiotic regimens [79,92–97]. Of high importance to their activity is the overall balance between lipophilicity and charge with the presence of two units of bulk and two cationic charges mandatory for membrane interaction and antimicrobial activity [16,78]. Naturally occurring antimicrobial peptides have a largely lipophilic character with the primary sequence consisting of 30–50% bulky hydrophobic residues such as tryptophan [26]. Tetra and pentapeptides possess a relatively small pharmacophore with limited bulk, thus in order to produce a favorable increase in the lipophilicity to charge ratio the amino terminus is acylated with a variety of hydrocarbon moieties [98]. With this knowledge Haug *et al.* produced a series of smaller dipeptides of a general formula XRY where; X represented a variant of a bulky amino acid with a lipophilic side chain, for example cyclohexylalanine; R represented arginine, the charged moiety; Y represented a C-terminal ester or a lipophilic amide derivative [79]. Both X and Y provided bulk to the short pharmacophore. A 2,5,7-tri-*tert*-butyl-tryptophan-arginine-benzyl amide was shown to be the most active peptide in the study. This structure correlates with the minimum requirement for two units of bulk, provided by the tryptophan derivative and benzene ring of the benzyl amide and two units of charge imparted by arginine’s guanidino group and the free N^α^-amino terminus. Central to this theory Bisht *et al.* produced a series of ornithine and tryptophan containing tetrapeptides and evaluated them against planktonic forms of various Gram-positive and Gram-negative bacteria [21]. To increase hydrophobicity cinnamic acid and its derivatives were attached to the amino terminus of the peptide motif. These terminal carboxylic acids included cinnamic acid, 3,4-dimethoxycinnamic acid, 4-hydroxy cinnamic acid and 3-(4-hydroxyphenyl) propionic acid.

### 1.7. Lipopeptides

Lipopeptides are essentially a peptide attached to a lipid moiety. Such a peptide may include an antimicrobial peptide. Native lipopeptides are formed in bacteria and fungi non-ribosomally by cultivation on a range of carbon sources to form complex cyclic structures [99]. They normally consist of a sequence of six or seven amino acids with a N-terminal fatty acid moiety attached. They are active against a range of multi-resistant bacteria and fungi [100]. Antimicrobial lipopeptides are primarily bacterial compounds synthesized via non-ribosomal biosynthetic pathways and comprise a peptidyl portion conjugated to a fatty acid to form an acylated peptide [99]. Many naturally occurring lipopeptides are cyclized and may also contain unnatural amino acids that confer stability against proteolytic degradation. Lipopeptides may comprise an anionic (e.g., Daptomycin, surfactin) or cationic (e.g., polymyxin B, colistin) peptide motif, which dictates the spectrum of activity [99]. Fatty acids, fatty amines, alcohols and gylceryl esters have all been shown to exhibit varying degrees of antimicrobial activity [101,102], whilst acylation of peptide scaffolds has been demonstrated to significantly improve antimicrobial activity [99,103]. For example when the natural occurring polymyxin is coupled to a fatty acid tail, removal of this tail leads to a decrease in antimicrobial action [104]. The conjugation of a fatty acid moiety at the N-terminus can also compensate for a loss of hydrophobicity within the peptidic chain based on amino acid residue selection [105]. Therefore, the combination of an optimized peptidyl scaffold and N-terminal acyl substituent (*i.e*., fatty acid) with inherent antimicrobial activities represents an approach to the development potent antimicrobial agents, whereby spectrum of activity may be modulated via modification of the N-terminal substituent, whilst circumventing the commercial barriers associated with the manufacture of natural antimicrobial peptides in high yields.

### 1.8. Licensed and Commercially Available Lipopeptides

A variety of anionic and cationic lipopeptides are currently licensed for commercial use, thus highlighting the clinical potential of these antimicrobials. These include daptomycin, polymyxin B and polymyxin E (colistin).

#### 1.8.1. Daptomycin

**Figure 1:**
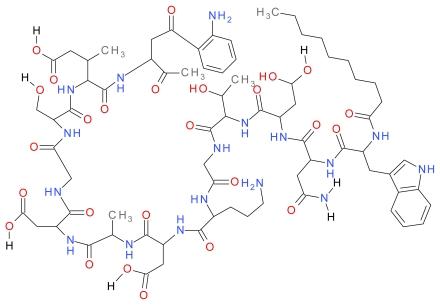
The structure of Daptomycin. Adapted from Steenbergen *et al*. 2005 [113].

Daptomycin is an anionic lipopeptide that is produced naturally by *Streptomyces roseosporus* (Actinobacteria). Daptomycin is a cyclic depsipeptide consisting of thirteen amino acids, which includes three D-amino acid residues (D-asparagine, D-alanine, and D-serine) linked to a hydrocarbon tail ten carbons in length derived from decanoic acid (Figure 1). Synthetic production of lipopeptides has led to the approval of the lipopeptide daptomycin which gained US and EU product licenses in 2003 and 2006 respectively for skin and soft tissue infections [106]. More recently it has been passed for use in the US for staphylococcal bacteremia and right sided endocarditis [107]. Daptomycin is also available in the UK as an injection and is commercially available as the brand Cubicin^®^ with further research showing possible use in the treatment of enterococcal bacteremia caused by *Enterococcus faecium* and *Enterococcus faecalis* [108]. Daptomycin, itself anionic, is dependent on the presence of calcium cations for antimicrobial activity. Electrostatic interactions allow daptomycin to alter to an active conformation. Increased amphiphilicity and integration of daptomycin into bacterial membranes occurs via the acyl chains [109–113]. This results in the formation of micelle-like structures with the lipid tails of daptomycin pointing inwards and the anionic side groups held together by calcium ions [114]. At bacterial membranes these micelles dissociate with daptomycin oligomerising in the membrane creating a potassium efflux ultimately resulting in; membrane disruption; cessation of the synthesis of the macromolecules Deoxyribonucleic acid (DNA), Ribonucleic acid (RNA) and protein synthesis; eventually leading to cell death. Unlike β-lactams this process is cell lysis independent with only the cell membrane, not the cell wall, disrupted [115]. Generally, daptomycin is well tolerated, although myopathy has been reported in some patients [116].

#### 1.8.2. Polymyxins (B and E)

Polymyxins are a group of cationic antimicrobials discovered in the late 1940s [117–119]. Polymyxin E was later identified in the 1950s [120]. Polymyxins are N-terminally fatty acylated cationic lipopeptides consisting of a cyclic peptide structure attached to a hydrocarbon tail. Removal of the fatty acid tail reduces the antimicrobial activity of polymyxin [104]. They are isolated naturally from the Gram-positive bacteria *Bacillus polymyxia* [121]. Only two polymyxins are commercially available, polymyxin B and polymyxin E (colistin) [122]. Polymyxin B is a cyclic heptapeptide with a tripeptide side chain that is acylated at its amino terminus by a fatty acid. Polymyxin B consists of the amino acids D-phenylalanine, l-threonine and l-α-γ-diaminobutyric acid [123,124]. Colistin has the same structure as polymyxin B but d-phenylalanine is replaced by d-leucine [124]. A range of topical and parenteral formulations are available indicated for the treatment of Gram-negative skin and eye infections, including those involving *Pseudomonas aeruginosa*, with a powdered nebulizer solution available to treat multidrug resistant Gram-negative infection in cystic fibrosis patients [125–127]. The mechanism of action of the polmyxins is similar to cationic antimicrobial peptides in general [128,129]. Interaction of the hydrophobic tail with lipopolysaccharides present on the outer membrane of Gram-negative bacteria creates a detergent-like effect compromising the integrity of the bacterial membrane [130]. Polymyxins may also displace magnesium and calcium ions from cationic binding sites present at the bacterial cell surface resulting in leakage of cell contents from the cytoplasm [131]. The membrane permeabilisation effect also has the benefit of making Gram-negative bacteria more susceptible to hydrophobic antimicrobials for example erythromycin [132–134]. Polymyxins also have the benefit of binding to and neutralizing Gram-negative endotoxins [135,136]. However the systemic use of polymyxins is limited with nephrotoxicity, ototoxicity and neurotoxicity common [137–139]. Colistin methanesulphonate, a prodrug of colistin, has shown increased use therapeutically due possessing a lower toxicity profile than colistin itself [140].

## 2. Mechanism of Action of Antimicrobial Peptides

### 2.1. Targeting of the Microbial Cell Membrane

As with the majority of antimicrobials, interaction with the cell membrane of microorganisms is fundamental to the mode of action of cationic antimicrobial peptides. The significant difference in the compositions of eukaryotic membranes in comparison to prokaryotic membranes highlights the important selectivity of cationic antimicrobials for bacterial cells. Phospatidylcholine, phosphatidylethanolamine, an analogue of phospatidylcholine, sphingomyelin together with the sterols, ergosterol and cholesterol are predominantly found in eukaryotes and normally have no net charge leading to an overall neutrally charged phospholipid bilayer. In comparison prokaryotic bacterial cytoplasmic membranes are negatively charged, with a high electrical potential gradient, contributed in part to the presence of acidic hydroxylated phospholipids such as phosphatidylglycerol, cardiolipin and phosphatidylserine [12]. Cationic antimicrobial peptides will therefore bind preferentially to the negatively charged phospholipid bilayer of bacterial cells [34,141]. This is advantageous with regard to reducing toxicity in any potential therapeutic environment. The lack of specific receptors will also make it difficult for bacteria to develop resistance to the peptide. Bacteria would have to alter the properties of their membrane as a whole rather than specific receptors. The overall positive charge associated with cationic antimicrobial peptides means that initial electrostatic interaction with the bacterial cell membrane will involve areas of dense anionic charge. Acidic polymers such as teichoic acids in Gram-positive [142] and phosphate groups present on lipopolysaccharides in Gram-negative bacteria [143] allow attachment of the peptide prior to formation of transmembrane pores and ultimately membrane permeabilization.

**Figure 2:**
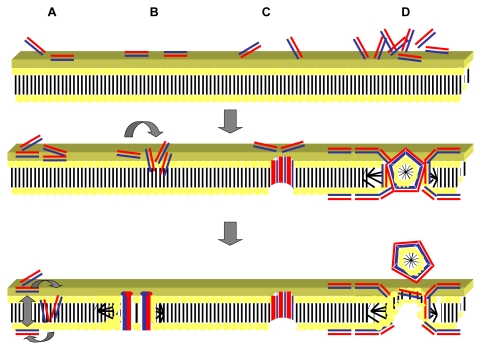
Proposed mechanisms of action of antimicrobial peptides. Antimicrobial peptides (cylinders) with the charged hydrophilic regions (red) and hydrophobic regions (blue). (**A**) The “aggregate” model: the antimicrobial peptides reorient to form an aggregate that spans the membrane, composed of peptide and lipid micelle complexes but with no particular orientation adopted; (**B**) The “toroidal pore” model occurs when the peptides insert perpendicular to the plane of the lipid bilayer, with hydrophilic groups on the peptide interacting with the membranous phospholipid head groups and the hydrophobic regions associating with the lipid core. A lipid bilayer lined pore is created as the membrane curves inwards; (**C**) The “barrel-stave” model also involves insertion of the peptides at a perpendicular orientation to the plane of the bilayer but staves are formed in a barrel shaped cluster due to hydrophilic portions of the peptide interacting with the lumen of the pore and hydrophobic regions of the peptide associating with the lipid bilayer; (**D**) The “carpet” model involves the aggregation of peptides at a parallel orientation to the lipid bilayer with localized carpeting of areas of the membrane. Micelles are formed above a critical threshold concentration leading to a detergent-like activity and the formation of pores in the membrane. Adapted from Jenssen, Hamill and Hancock 2006 [147].

Fungal cell membranes tend to share similar properties to the zwitterionic eukaryotic membranes [144]. Peptides that primarily possess antifungal activity tend to consist of neutral amino acids with regions of high polarity suggesting that a unique structure-activity relationship exists [145]. Proof of this structure activity relationship and the importance of lipophilic moieties in the peptide structure were provided by Lopez-Garcia *et al.* [146]. They demonstrated that there was a direct correlation between the antifungal activity of peptides and the ability of peptides to form aggregative complexes with lipid formulations. Four main hypotheses exist as to how antimicrobial peptides achieve entry into the bacterial cell (Figure 2: part A–D). These are the toroidal pore, aggregate, barrel stave, and carpet models.

The toroidal pore model involves the peptide inserting perpendicular into the cell membrane *via* electrostatic interactions between the hydrophilic regions of the peptide and the phospholipid head of the bilayer. Hydrophobic regions of the peptide bend the lipid monolayers forming a central water core lined by lipid head groups and inserted peptides [148]. In doing this the membrane bends inwards such that the bilayer lines the channel as well as the peptides. A toroidal pore is thus formed by positive curvature, allowing entry of further antimicrobial peptide [149]. In the aggregate model a similar process occurs to that of the toroidal pore model. However, peptides do not adopt any specific orientation upon insertion into the membrane but cover the membrane as an aggregate of peptide and lipid micelles [150]. Channels that do form vary greatly, so much that partial membrane insertion may lead to the formation of negative curvature and peptide aggregation within the bilayer [151].

Perpendicular insertion of the peptides forming barrel-like clusters or staves occurs in the barrel-stave model [152]. Pores can occur from as little as three peptide molecules and theoretically to allow these pores to form, peptides must have an amphipathic or hydrophobic α-helix, β-sheet structure or both [153]. Usually the barrel-stave model results in a transmembrane pore of unilateral size. Within this pore hydrophilic regions of the peptide oppose the lumen, forming the interior and Van der Waal’s attractions occurring between the hydrophobic peptide regions and the lipid core [154]. The carpet model suggests that peptides accumulate parallel to the membrane with the hydrophobic regions of the peptide associating with the anionic phospholipid head groups on the membrane surface and hydrophilic regions attracted to the polar solvent [155]. Localization of peptides occurs forming a carpet-like coating on the membrane until a threshold concentration is reached. At this threshold a detergent-like process occurs with the eventual formation of micelles and transient pores. Disruption of the membrane structure leads to membrane disintegration [156]. It has been hypothesized that the additional outer lipid membrane present on Gram-negative organisms consisting of lipopolysaccharides allows a self-promoted uptake pathway to occur for cationic peptides [157,158]. As the majority of these peptides have a high affinity for lipopolysaccharides they bind to them, competitively replacing divalent cations such as magnesium and calcium ions from their relative binding sites [159]. Both magnesium and calcium ions are required for cell surface stability via the cross-linking of carboxylated and phosphorylated head groups of lipids [160]. Removal of these divalent cations leads to distortion of the outer membrane forming holes through which further peptide and other small molecules (such as conventional antibiotics) can cross. The self-promoted uptake model provides an explanation as to why in Gram-negative bacteria many cationic antimicrobial peptides act in synergy with conventional antibiotics [161].

Synergy of cationic antimicrobial peptides with standard antimicrobials is not limited to just Gram-negative bacteria but has been proven for both Gram-positive [162] and fungi also [163]. A similar pattern of membrane disruption has been demonstrated for antimicrobial peptides against a range of fungal pathogens. The rabbit α-defensin NP-2, magainin-2 and bovine lactoferrin have been shown to cause membrane permeabilization and cell wall damage in *Candida albicans* [164–166]. The antimicrobial lipopeptide iturin, obtained from cultures of *Bacillus subtilis*, is fungicidal through its activity on cell membranes [167]. Lipopeptide aggregates and lipopeptide/phospholipid complexes form at the peptide-membrane interface resulting in the creation of ionic pores that allows the increased influx of potassium ions and fungal cell death [168]. These pores may also provide a means by which ATP is released from damaged cells. Membrane damage results in leakage and localized increases in extracellular ATP as the microbial membrane is damaged but intracellular metabolic processes continue. Vylkova *et al.* hypothesized that this may contribute to cell death in *Candida albicans* and other microorganisms by facilitating further peptide uptake or activating the host’s innate immune response with ATP acting as a chemo-attractant at the site of infection [169].

### 2.2. Mechanism of Action of Antimicrobial Peptides: Intracellular Targeting

**Figure 3:**
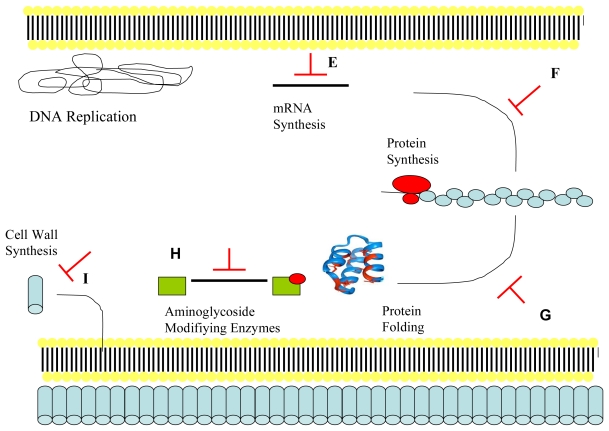
The intracellular action of some antimicrobial peptides (E−I). (**E**) Dermaseptin, buforin-II and pleurocidin are antimicrobial peptides that have been shown to inhibit both DNA and RNA synthesis at MIC values [188]. Dermaseptin inhibits RNA synthesis in bacteria at MIC concentration or higher within 5 minutes, with lack of bactericidal action within 30 minutes in *Escherichia coli* evidence of a mainly intracellular action [189]. Buforin-II contains a single proline residue within its primary structure that allows translocation across cell membranes without membrane destruction, with binding to nucleic acids resulting in cell death [170,190]; (**F**) PR-39 and indolicidin have been shown to inhibit the rate of protein synthesis and is therefore a plausible target for antimicrobial peptides [13,180,191]; (**G**) Pyrrhocoricin and drosocin act one step later than the molecules of part E (dermaseptin, buforin-II and pleurocidin) and have been shown to reduce enzymatic activity via inhibition of ATPase activity of the heat-shock protein DnaK, an enzyme involved in chaperone-assisted protein folding [192–194]. Otvos and colleagues formed a chimeric dimer, with newly formed activity against *Staphylococcus aureus* and increased activity against previously sensitive *Escherichia coli*, by synthesizing a molecule that possessed pyrrhocoricin’s DnaK binding domain and drosocin’s high membrane permeating ability [195]; (**H**) Antimicrobial peptides may also inhibit resistance mechanisms linked to bacterial pathogenesis for example enzymes with anionic binding site pockets linked to the modification of aminoglycoside antibiotics [171]; (**I**) Lantibiotics such as mersacidin and nisin target the formation of structural components of the cell wall, specifically the transglycosylation of lipid II, necessary for the synthesis of peptidoglycan [196,197]. Adapted from Jenssen, Hamill and Hancock 2006 [147].

Membrane damage is only one of many mechanisms that antimicrobial peptides may possess in exerting microbial cell death. In many cases it may not be the principle mechanism. There is increasing evidence for intracellular targeting of microbes (Figure 3: part E–I) as both alternative and synergistic pathways to membrane rupture and cell lysis. The antimicrobial peptide buforin II, possessing a linear proline hinge and containing an amphipathic α-helical peptide, has been proven to translocate across the cell membrane without loss of the transmembrane potential and with intracellular contents intact even at five times the MIC. Cellular function of *Escherichia coli* is inhibited by accumulation of buforin II in the cytoplasm and via binding to DNA and RNA [170]. Binding to and inhibition of cellular nucleic acids by cationic antimicrobial peptides is feasible due to the polyanionic charges present in nucleic acids and also some intracellular enzymes [26,171].

Some antimicrobial peptides such as indolicidin have been demonstrated to penetrate bacterial cell membranes rendering them relatively undamaged but with antibacterial activity achieved by inhibition of RNA, DNA and protein synthesis [172]. This contrasts to a mainly membrane active targeting of fungal cells by indolicidin via direct interactions with the phospholipid bilayer [173]. Other hypotheses for intracellular action include stimulation of the autolytic enzyme cascade [174]. Cationic antimicrobial peptides, for example lactoferrin and lysozyme, may mimic the action of β-lactam antibiotics and activate autolytic cell wall enzymes such as muramidases causing bacteriolysis [175]. Intracellular targeting of protein synthesis by degradation of proteins required for DNA replication has been shown to be a primary mechanism of action for indolicidin and the pig intestinal peptide PR-39 [176–178]. Indolicidin and lactoferricin B have also been shown to induce filamentation, a process in which bacterial cells continue to elongate but cannot divide as the septum does not develop, and significant cell lysis [179–181]. Filamentation results from inhibition of DNA synthesis [182].

In mammals the antimicrobial cathelicidin peptides LL-37 and PR-39 have multiple membrane and intracellular mechanisms of action but they can also mitigate the immune response to foreign pathogens [183,184]. LL-37 induces the selective movement of neutrophils, monocytes and CD4 T-lymphocyte cells allowing an increase in the adaptive and innate immune response [185,186]. The porcine peptide PR-39 promotes healing via stimulation of angiogenesis mediated by its degradation of hypoxia inducible factor 1a protein [187].

Alternative bactericidal intracellular mechanisms are outlined in Figure 3: parts E-I. By possessing multiple microbial targets, antimicrobial peptides can be a therapeutically valuable tool as the potential for microbial resistance is low (perhaps negligible). The chances of resistance developing is lessened further by many antimicrobial peptides possessing similar MIC and minimum bactericidal concentration (MBC) values (MBC no more than double the value obtained for MIC), owing to mainly bactericidal action [198]. The intracellular mode of action of antimicrobial peptides against fungal pathogens is linked to interactions with the fungal mitochondrion, with targeting and perturbation of the mitochondrial membrane shown by Helmerhorst *et al.* [199]. The cationic peptides histatins, derived from the saliva of humans, bind to receptors present on the fungal cell membrane and when present in the cytoplasm they target fungal mitochondria [200]. The echinocandins are an antimicrobial peptide family sourced from *Aspergillus nidulans* that includes the peptides anidulafungin caspofungin, and micafungin [201]. They have been shown to inhibit β-(1,3)-glucan synthase, an enzyme responsible for the production of the essential fungal cell wall component β-(1,3)-glucan [202–204].

## 3. Development of Resistance to Antimicrobial Peptides

The multiple modes of action utilized by antimicrobial peptides reduces the ability of microorganisms to develop resistance, with cidal activity also shown against bacteria resistant to standard antibiotics [205]. The formation of a highly hydrated extracellular polymeric phenotype or biofilm contributes to antimicrobial resistance by blocking the transport of antimicrobials through the biofilm matrix. Possible mechanisms for this to occur are by binding of the biofilm to them directly, as in the case of positively charged aminoglycoside antibiotics, restricting their permeation and by restricting diffusion of larger antimicrobials [206,207].

**Figure 4:**
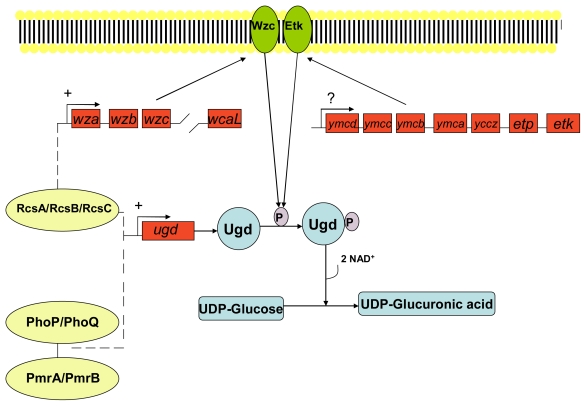
The synthesis of Undecaprenyl phosphate-glucuronic acid via quorum sensing systems. The two component quorum sensing systems PhoP/PhoQ and PmrA/PmrB; or the RcsA/RcsB/RcsC system alone allow the expression of genes (*ugd*) involved in the intracellular synthesis of the 4-amino-4-deoxy-L-arabinose precursor: Undecaprenyl phosphate-glucuronic acid. Both scenarios involve the phosphorylation of the protein Ugd by the membrane bound autophosphorylated protein-tyrosine kinase Wzc and/or Etk. Etk and Wzc are a BY-kinase (a newly defined group of enzymes involved in protein-tyrosine phosphorylation) of *Escherichia coli* and are involved in the production of the group IV capsule surrounding the cell membrane [209]. The *wca* operon is upregulated by the RcsA/RcsB/RcsC system and consists of 19 genes with the third gene in order of transcription being the *wzc* gene that has been shown to encode a membrane bound autophosphorylated protein-tyrosine kinase Wzc [210]. Etk is coded for by the *etk* gene present on the *ymc* operon of some pathogenic strains of *Escherichia coli* [211]. The mechanism of the *ymc* operon is itself unknown, although it could possibly be a promoter of *etk* expression. Key: UDP: Undecaprenyl phosphate. (Adapted from Lacour, 2008 [212])

**Figure 5:**
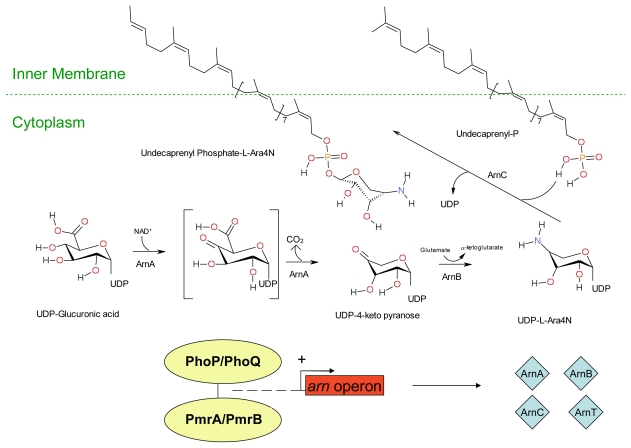
The synthesis of Undecaprenyl Phosphate-α-4-amino-4-deoxy-L-arabinose from Undecaprenyl-Glucuronic acid. The enzymes ArnA, ArnB, ArnC and ArnT are produced by transcription of the *arn* operon which is upregulated by the RcsA/RcsB/RcsC and PmrA/PmrB quorum sensing systems. The enzyme ArnA catalyses the oxidative decarboxylation of Undecaprenyl -glucuronic acid, thus forming an Undecaprenyl -4-keto-pyranose intermediate. ArnC catalyzes the transfer of the 4-amino-4-deoxy-L-arabinose moiety to Undecaprenyl phosphate in the inner membrane, forming the Undecaprenyl phosphate-α-4-amino-4-deoxy-L-arabinose. Key: UDP: Undecaprenyl phosphate. (Adapted from Lacour, S. 2008 [212]).

**Figure 6:**
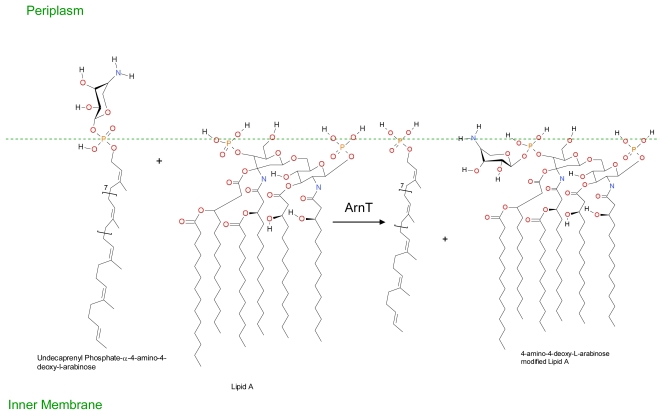
The transfer of 4-amino-4-deoxy-L-arabinose moiety to Lipid A. Translocation to the outer surface of the inner membrane occurs via an unknown mechanism. ArnT transfer the 4-amino-4-deoxy-L-arabinose moiety from Undecaprenyl phosphate-α-4-amino-4-deoxy-L-arabinose to Lipid A, thereby reducing the affinity of Lipid A for polymyxin and other cationic antimicrobial peptides. (Adapted from Lacour, S. 2008 [212]).

Extracellular DNA may also play a role in increased resistance of biofilm forms of *Pseudomonas aeruginosa* against cationic antimicrobial peptides. Extracellular DNA is a cation chelator and acts to sequester cations from the surrounding environment and also plays a role in the modification of the cationic antimicrobial peptide binding site lipid A by the sugar dehydrogenases enzyme Undecaprenyl phosphate-glucose dehydrogenase and covalent binding to 4-amino-4-deoxy-L-arabinose [208]. Resistance is conferred via covalent modification of the cationic antimicrobial binding site lipid A. Lipid A is a hydrophobic anchor situated on the outer surface of the inner membrane of Gram-negative bacteria and acts as an anionic membrane target for cationic drugs such as polymyxin and also cationic antimicrobial peptides. Covalent binding to 4-amino-4-deoxy-L-arabinose moiety reduces both the anionic charge of lipid A and its affinity for cationic antimicrobials. In Gram-negative bacteria such as *Escherichia coli* the expression of genes (*arn* and *ugd* operons) involved in Undecaprenyl phosphate-4-amino-4-deoxy-L-arabinose production are controlled by the two component quorum sensing systems PhoP/PhoQ and PmrA/PmrB; and/or the RcsA/RcsB/RcsC system as outlined in Figures 4, 5 and 6.

## 4. Future Perspectives

As biocides antimicrobial peptides have the potential to eradicate the most resistant forms of clinically relevant biofilm forming pathogens. Lipopeptides such as polymyxin B and daptomycin are already utilized in topical formulations [213], therefore the potential exists for the ornithine and tryptophan containing peptides to be exploited similarly. More advanced forms of topical treatment would mimic the use of the polycationic lipopeptide colistin and the polycationic trisaccharides tobramycin and gentamicin as aerosol therapy for the treatment of persistent lung infections in cystic fibrosis patients [214,215]. The potential for natural and synthetic peptides as therapeutic molecules go beyond the boundaries of microbial biofilm infection. Research has extended to the use of these peptides from areas as diverse as cancer treatment [216], to the eradication of sexually transmitted diseases [217], such as HIV [218], with a dual role as an effective contraceptive spermicide [219,220]. A group of cationic peptides, referred to as cell-penetrating peptides can translocate into the cell cytoplasm without disruption of the cell membrane. Peptides such as Apidaecins have the potential to act as precursors for the transport of alternative drugs to mammalian cells [221]. Despite the fact there are thousands of naturally sourced antimicrobial peptides and millions of potential synthetic possibilities there have been limited clinical trials based on antimicrobial peptides [222]. Only a relative few, for example daptomycin, have entered into clinical trials and therapeutic use based on *in vitro* results and animal studies [106,223,224]. Issues still remain with regard to the stability of peptide based formulations *in vivo* and the large scale production costs of these peptides. It is expected that future research will allow the area of antimicrobial peptides to be harnessed therapeutically with the same degree of evolutionary success as they are utilized in nature as components of innate immunity. Antimicrobial peptides fulfill a number of the criteria expected from an ‘ideal’ biocide, namely performance (high cidal activity and a rapid rate of kill across a range of microorganisms), environmental fate (facile bioremediation), safety and cost.
